# Comparative evaluation of FIB-3 and FIB-4 indices for liver fibrosis screening in workplace-based health checkups

**DOI:** 10.1093/joccuh/uiaf038

**Published:** 2025-07-09

**Authors:** Kota Fukai, Shoko Nakazawa, Kosuke Sakai, Yuko Furuya, Yuya Watanabe, Toru Honda, Takeshi Hayashi, Toru Nakagawa, Masaaki Korenaga, Masayuki Tatemichi

**Affiliations:** Department of Preventive Medicine, Tokai University School of Medicine, Isehara, Japan; Department of Preventive Medicine, Tokai University School of Medicine, Isehara, Japan; Department of Preventive Medicine, Tokai University School of Medicine, Isehara, Japan; Department of Preventive Medicine, Tokai University School of Medicine, Isehara, Japan; Hitachi Health Care Center, Hitachi, Japan; Hitachi Health Care Center, Hitachi, Japan; Occupational Hygiene and Promotion Center, Hitachi Ltd, Tokyo, Japan; Hitachi Health Care Center, Hitachi, Japan; Hepatitis Information Center, The Research Center for Hepatitis and Immunology, National Institute of Global Health and Medicine, Japan Institute for Health Security, Ichikawa, Japan; Department of Preventive Medicine, Tokai University School of Medicine, Isehara, Japan

**Keywords:** Fib-3 index, Fib-4 index, liver fibrosis, steatotic liver disease, noninvasive screening, workplace health checkups, negative predictive value

## Abstract

**Objectives:**

To examine the utility of the FIB-3 index as a secondary screening tool for liver fibrosis in workplace-based health checkups, by comparing its concordance and negative predictive values (NPVs) with those of the FIB-4 index.

**Methods:**

This cross-sectional study included 12 622 workers from the Hitachi Cohort Study who underwent workplace-based health checkups between April 2021 and March 2022. FIB-4 was calculated using age, aspartate aminotransferase (AST), alanine aminotransferase (ALT), and platelet count, whereas FIB-3 used the same components except age. To evaluate the utility of FIB-3 in excluding liver fibrosis, NPVs were calculated using FIB-4 thresholds (1.30, 2.01, and 2.67) as references. Concordance between FIB-3 and FIB-4 was examined across different age groups. In addition, multivariate logistic regression analysis was conducted to identify factors associated with false-positive FIB-3 results.

**Results:**

The FIB-3 index demonstrated high NPVs for excluding liver fibrosis, with values of 99.9% at FIB-4 ≥ 1.30 and 98.2% at FIB-4 ≥ 2.67. Strong concordance between FIB-3 and FIB-4 was observed consistently across different age groups. Among participants with elevated ALT (>30 IU/L), FIB-3 consistently ruled out fibrosis, whereas FIB-4 positivity increased with advancing age. Multivariate analysis indicated that higher AST levels and increased alcohol intake were significantly associated with false-positive FIB-3 results.

**Conclusions:**

The FIB-3 index demonstrated stable performance across age groups while maintaining high concordance and NPV relative to FIB-4. These findings suggest that FIB-3 may serve as a practical screening tool in routine workplace-based health checkups, particularly in mitigating age-related overestimation observed with the FIB-4 index.

## Teaser

Key points

**What is already known on this topic:** FIB-4 is a widely used noninvasive index for liver fibrosis screening and has a high negative predictive value, but its inclusion of age can result in false positives among older individuals. There is a growing need for age-independent markers like FIB-3 in workplace health screenings.

**What this study adds:** This study showed that FIB-3 maintains a high negative predictive value and strong concordance with FIB-4 across age groups in a large cohort of workers. It effectively excludes fibrosis in individuals with elevated alanine aminotransferase and avoids the age-related inflation observed with FIB-4. Factors such as elevated aspartate aminotransferase and alcohol consumption were associated with false-positive FIB-3 results.

**How this study might affect research, practice, or policy:** FIB-3 may reduce unnecessary referrals for older workers and support more efficient liver screening during routine occupational health checkups. These findings encourage incorporating FIB-3 and platelet counts into standard health assessments and call for further validation with pathological data.

## 1. Introduction

Global concern regarding liver disease has shifted from viral hepatitis to a spectrum of liver diseases, including steatotic liver disease, liver fibrosis, and hepatocellular carcinoma.^1^ This shift was driven by a decline in hepatocellular carcinoma cases and liver-related deaths caused by viral hepatitis.^2^ Estes et al^3^ predicted that the prevalence of steatotic liver disease, including nonalcoholic steatohepatitis, will increase by 15%-56% worldwide, with liver-related mortality more than doubling in specific populations by 2030. Reflecting this trend, the 2023 Delphi consensus replaced nonalcoholic fatty liver disease/nonalcoholic steatohepatitis with steatotic liver disease.^4^ The Japan Society of Hepatology has raised awareness of steatotic liver disease and issued the “Nara Declaration 2023,”^5^ recommending that individuals with alanine aminotransferase (ALT) levels of ≥30 IU/L consult their physician. However, following this recommendation in the context of mandatory annual health checkups for workers, over 15% of male workers with ALT levels of ≥40 pose challenges owing to concerns over labor loss and medical costs.^6^

To address the growing concern of liver disease in workplaces where viral hepatitis countermeasures are implemented, one potential approach for employees with ALT levels of ≥30 IU/L is secondary screening using fibrosis markers before recommending a visit to their family doctor. The FIB-4 index (FIB-4), calculated using age, ALT, aspartate aminotransferase (AST), and platelets, is widely used in clinical settings and is known for its high negative predictive value (NPV).^7^^,^^8^ A FIB-4 score below the standard threshold can effectively rule out liver fibrosis with 99% certainty.^9^ However, there are challenges in using FIB-4 in workplace settings, mainly because the formula incorporates age, which can inflate the FIB-4 scores in older individuals. Although attempts have been made to establish age-specific cutoffs, the complexity of such adjustments limits their practical applications. Furthermore, in Japan’s mandatory health checkups, red blood cell counts and hemoglobin are required by law. Platelet counts, although routinely measured, are often not used because they are not legally mandated to be included in health checkup reports. These data are frequently omitted or intentionally deleted, despite their potential usefulness in assessing liver fibrosis.^10^ Integrating platelet counts into routine health checkup analyses could enable the widespread use of fibrosis markers like FIB-4 and FIB-3, which rely on platelet data, thereby improving the detection of liver fibrosis in the working population. As the workforce ages, there is an increasing need for age-independent fibrosis markers that can be fully utilized, including those based on platelet counts.

**Figure 1:**
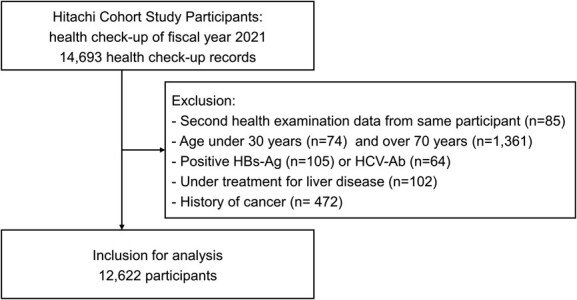
Flow diagram of the study. Abbreviations: HBs-Ag, hepatitis B virus surface antigen; HCV-Ab, antibody to hepatitis C virus antigen.

Recently, Kariyama et al^11^ proposed the FIB-3 index (FIB-3), which excludes age and offers a potential alternative to FIB-4; its effectiveness has been demonstrated by early studies. Nouso et al^12^ reported that FIB-3 is an improved version of FIB-4 capable of predicting steatotic liver disease associated with liver fibrosis metabolic dysfunction. However, FIB-3 was developed primarily for clinical use and has yet to be thoroughly evaluated in a workplace-based population setting. Given the growing need for efficient, noninvasive fibrosis screening in large-scale health checkups, further evaluation of the role of FIB-3 is critical. Building on our previous findings regarding FIB-4 in routine health examinations,^10^^,^^13^ herein we provide evidence for the practical application of FIB-3 in large-scale health screening programs.

In this study, we assessed the utility of FIB-3 as a secondary screening tool by examining its concordance with FIB-4 and evaluating its NPV in individuals with elevated ALT levels. We also sought to identify the factors contributing to the discrepancies between FIB-3 and FIB-4, emphasizing the need to incorporate platelet counts in routine health checkups, where they are often measured but not utilized.

## 2. Methods

### 2.1. Study design and participants

This cross-sectional study used data from the Hitachi Cohort Study conducted at the Hitachi Health Care Center in Ibaraki, Japan. The cohort consisted of employees from approximately 30 affiliated companies who have participated in annual health checkups since April 1, 2000. Data collection and management were conducted as previously described.^10^^,^^13^^,^^14^ Health examination data were collected on various anthropometric and biochemical parameters and information on disease history and lifestyle habits.

For the present analysis, 14 693 health checkup records from the mandatory health checkups conducted during the fiscal year 2021 (April 1, 2021 to March 31, 2022) were analyzed. We excluded participants who had secondary health examination data in the same fiscal year (*n* = 85), those aged less than 30 years (*n* = 74) and over 70 years (*n* = 1361), those who tested positive for hepatitis B virus surface antigen (*n* = 74) or antibody to hepatitis C virus antigen (*n* = 64), those undergoing treatment for any liver disease (*n* = 102), and those with a history of cancer (*n* = 472). We excluded participants with a present or past history of malignancy since some anticancer drugs may have affected the platelet counts.^15^ The participant selection process is illustrated in Figure 1.

All participants were informed of the study objectives and procedures via email, intranet, or their company’s Committee of Occupational Health and Safety. Employees who provided informed consent were included in the final dataset. No participants declined or withdrew their consent after being informed of the study. The study was conducted in accordance with the ethical guidelines outlined in the Declaration of Helsinki and conformed to the Strengthening the Reporting of Observational Studies in Epidemiology (STROBE) checklist (see Table S1),^16^ a guideline used to improve the quality of observational study reports. The Institutional Review Board for Clinical Research of Tokai University (approval number: 20R369) and the Hitachi Review Board (approval number: 2021-16) approved this study.

**Table 1:** Characteristics of participants by ALT values.^a^

	**ALT ≤30**		**ALT >30**		**Total**	
Variable	*n* or mean	% or SD	*n* or mean	% or SD	*n* or mean	% or SD
Number of participants	8999	(71.3)	3623	(28.7)	12 622	(100)
Male	7508	(83.4)	3470	(95.8)	10 978	(87.0)
Age, y	52.4	±9.3	50.4	±9.1	51.8	±9.3
BMI, kg/m^2^	23.4	±3.1	26.5	±4.2	24.3	±3.7
Waist, cm	82.5	±8.9	91.1	±10.3	85.0	±10.1
Systolic BP, mmHg	122.0	±12.9	125.7	±11.5	123.0	±12.6
Diastolic BP, mmHg	77.5	±9.0	80.7	±8.6	78.4	±9.0
Fasting blood glucose, mg/dL	105.7	±18.3	113.0	±24.8	107.8	±20.6
HbA1c, %	5.7	±0.6	5.9	±0.8	5.7	±0.7
HDL cholesterol, mg/dL	61.5	±15.8	53.7	±13.7	59.3	±15.6
LDL cholesterol, mg/dL	121.8	±28.9	128.2	±31.1	123.6	±29.7
Triglycerides, mg/dL	111.5	±79.2	163.9	±124.3	126.5	±97.3
AST, IU/L	20.0	±4.8	33.3	±15.1	23.8	±10.9
ALT, IU/L	18.9	±5.6	50.7	±24.7	28.0	±20.1
GGT, IU/L	33.6	±30.4	77.6	±80.6	46.3	±54.0
Current smoker	2679	(29.8)	1274	(35.2)	3953	(31.3)
Current drinker	5794	(64.4)	2356	(65.0)	8150	(64.6)
Alcohol intake, g/wk	33.1	±42.4	34.3	±43.3	33.4	±42.7

Abbreviations: ALT, alanine aminotransferase; AST, aspartate aminotransferase; BMI, body mass index; BP, blood pressure; GGT, γ-glutamyl transferase; HbA1c, glycated hemoglobin; HDL, high-density lipoprotein; LDL, low-density lipoprotein.

a Data are presented as numbers (percentages) for categorical variables and means ± SDs for continuous variables.

**Table 2:** Prevalence (%) exceeding the cutoff values for FIB-4 and FIB-3 by age class.

**Population**	**Age class**				
**Cutoff value**	**30s**	**40s**	**50s**	**60s**	**Total**
Total (*n* = 12 622)					
FIB-4 = 1.30	1.1	5.7	21.9	56.9	23.0
FIB-4 = 2.01	0.1	0.5	2.4	12.0	3.8
FIB-4 = 2.67	0.0	0.2	0.7	2.7	0.9
FIB-3 = 3.41	0.3	0.9	1.5	2.2	1.3
ALT >30 (*n* = 3623)					
FIB-4 = 1.30	0.4	6.2	24.0	58.0	21.1
FIB-4 = 2.01	0.0	0.8	3.8	15.6	4.4
FIB-4 = 2.67	0.0	0.4	1.6	5.8	1.7
FIB-3 = 3.41	0.4	2.1	3.7	7.2	3.3

Abbreviation: ALT, alanine aminotransferase.

### 2.2. Assessment of FIB-3 and FIB-4 indices

Blood tests were performed in the morning after overnight fasting. Serum liver function tests, including ALT, AST, and γ-glutamyl transferase (GGT), were performed using an autoanalyzer (Hitachi 7600; Hitachi Co. Ltd, Tokyo, Japan) following the Japan Society of Clinical Chemistry’s transferable national standardized method. Platelet counts were measured using an automated hematology analyzer, following the standardized procedures recommended by the Japan Society of Hematology.

FIB-4 was calculated using the following formula:

FIB-4 = (age [years] × AST [IU/L])/(platelets [10^9^/L] × √ALT [IU/L]).

The cutoff values for FIB-4 of 1.30, 2.01, and 2.67 were considered low, moderate, or high risk for liver fibrosis, respectively.^12^^,^^17^

FIB-3 was calculated using the following formula:

FIB-3 = 5 × ln (AST [IU/L]) − 2 × ln (ALT [IU/L]) − 0.18 × (platelet [10^4^/μL] − 5.

The cutoff value for the FIB-3 index of 3.41 was considered a risk factor for liver fibrosis, according to previous reports.^11^^,^^12^

### 2.3. Other variables

Height and weight were measured using standardized procedures.^18^ Body mass index was calculated by dividing the weight in kilograms by the height in meters squared. Waist circumference was measured using a standardized procedure, with measurements recorded to the nearest 0.1 cm. Medical personnel measured systolic and diastolic blood pressures (mmHg) according to standard protocols. The lowest blood pressure value was adopted if more than one measurement was obtained. Fasting plasma glucose levels were measured using the glucose oxidase enzyme electrode method (A&T, Tokyo, Japan). Glycated hemoglobin (HbA1c) levels were measured using high-performance liquid chromatography (HLC723-G9; TOSOH, Tokyo, Japan). The value for HbA1c (%) was estimated as the National Glycohemoglobin Standardization Program equivalent value (%), calculated using the formula HbA1c (%) = HbA1c (Japan Diabetes Society) (%) + 0.4%.^19^ Other blood tests, including high-density lipoprotein cholesterol, low-density lipoprotein cholesterol, and triglyceride levels, were performed using enzymatic methods. Questionnaires were used to assess alcohol consumption and smoking status. Total alcohol intake (grams ethanol per week) was calculated based on the reported frequency and quantity of alcohol consumed.^20^

**Figure 2:**
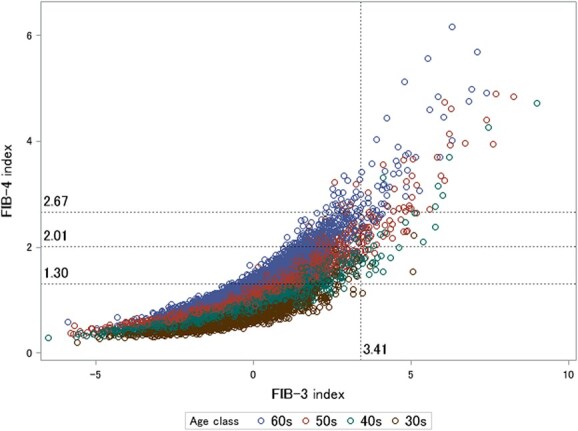
Scatter plot of FIB-4 versus FIB-3 across age groups and association with the cutoff thresholds (*n* = 12 622). Each data point represents an individual observation point. The points are color-coded to indicate the age groups of the participants: blue indicates individuals in their 30s, green indicates those in their 40s, brown indicates individuals in their 50s, and red indicates those in their 60s.

**Table 3:** Agreement for different FIB-4 cutoffs compared with FIB-3 ≥ 3.41 by age class among participants with ALT >30 (*n* = 3623).

		**FIB-4 ≥ 1.30**					**FIB-4 ≥ 2.01**					**FIB-4 ≥ 2.67**				
		**(−)**	**(+)**	**PABAK** ^**a**^	**AC1** ^**a**^	**NPV,** ^**b**^ **%**	**(−)**	**(+)**	**PABAK** ^**a**^	**AC1** ^**a**^	**NPV,** ^**b**^ **%**	**(−)**	**(+)**	**PABAK** ^**a**^	**AC1** ^**a**^	**NPV,** ^**b**^ **%**
Age class	FIB-3 ≥ 3.41	*n*	*n*	(95% CI)	(95% CI)		*n*	*n*	(95% CI)	(95% CI)		*n*	*n*	(95% CI)	(95% CI)	
30s	(−)	507	1	0.99 (0.98-1.00)	1.00 (0.99-1.00)	99.8	508	0	—	—	99.6	508	0	—	—	99.6
	(+)	1	1				2	0	—	—		2	0	—	—	
40s	(−)	1034	45	0.92 (0.90-0.94)	0.96 (0.94-0.97)	100.0	1079	0	0.97 (0.96-0.99)	0.99 (0.98-0.99)	98.7	1079	0	0.97 (0.95-0.98)	0.98 (0.97-0.99)	98.3
	(+)	0	23				14	9				19	4			
50s	(−)	1055	282	0.59 (0.55-0.64)	0.73 (0.70-0.77)	100.0	1331	6	0.98 (0.97-0.99)	0.99 (0.99-1.00)	99.6	1336	1	0.96 (0.94-0.97)	0.98 (0.97-0.98)	97.8
	(+)	0	51				5	46				30	21			
60s	(−)	262	316	−0.01	0.10 (0.01-0.18)	100.0	526	52	0.83 (0.79-0.88)	0.90 (0.87-0.92)	100.0	575	3	0.95 (0.93-0.98)	0.97 (0.96-0.99)	98.0
	(+)	0	45	(−0.09 to 0.06)			0	45				12	33			
Total	(−)	2858	644	0.64 (0.62-0.67)	0.77 (0.75-0.79)	99.9	3444	58	0.96 (0.95-0.97)	0.98 (0.97-0.98)	99.4	3498	4	0.96 (0.95-0.97)	0.98 (0.98-0.99)	98.2
	(+)	1	120				21	100				63	58			

Abbreviations: AC1, Gwet AC1 statistic; ALT, alanine aminotransferase; NPV, negative predictive value; PABAK, prevalence-adjusted bias-adjusted kappa.

a PABAK accounts for bias and prevalence effects, whereas AC1 is a robust alternative less affected by prevalence and marginal distribution imbalances. Both statistics range from 0 (no agreement beyond chance) to 1 (perfect agreement), with commonly used interpretation thresholds similar to those of Cohen kappa.

b NPVs were calculated as the ratio of individuals with FIB-3 > 3.41 to those with FIB-4 values under the threshold.

### 2.4. Statistical analysis

The characteristics of the participants are expressed as means (SD) and percentages for continuous and categorical variables, respectively. The prevalence of exceeding the cutoff values for FIB-4 and FIB-3 was calculated using 10-year age classes.

Cross-tabulations of the cutoff values for FIB-3 and FIB-4 were performed for each 10-year age group. Concordance between FIB-3 and FIB-4 was assessed using prevalence-adjusted bias-adjusted kappa (PABAK)^21^ and the Gwet AC1 statistic (AC1),^22^ with 95% CIs accounting for prevalence and bias. Additionally, to evaluate the performance of FIB-3 in predicting fibrosis, the NPV of FIB-3 was calculated by assuming a true fibrotic state for FIB-4 values greater than 1.3, 2.01, or 2.67. NPV was defined as the proportion of individuals with FIB-3 <3.41 among those with FIB-4 values below the specified thresholds.

To identify factors associated with false-positive FIB-3 results (FIB-3 ≥ 3.41) despite negative FIB-4 outcomes (FIB-4 < 2.67), multivariate logistic regression was employed. Adjusted beta coefficients and odds ratios (ORs) were calculated for each characteristic. For the sensitivity analysis, all analyses were conducted for the total population, the subset of participants with ALT levels >30 IU/L, and separately for women to evaluate the potential impact of sex on the findings.

Alpha was set to .05, and all *P* values were 2-sided. All statistical analyses were performed using the Statistical Analysis System (SAS) software V.9.4 (SAS Institute, Cary, North Carolina, USA).

## 3. Results

A total of 12 622 participants were included in the final analysis, of whom 28.7% (*n* = 3623) had ALT levels >30 IU/L. The characteristics of the participants according to their ALT levels are given in Table 1. The proportion of male participants was higher in the ALT >30 group than in the ALT ≤30 group (95.8% vs 83.4%, respectively). Participants in the ALT >30 group were generally younger (mean age 50.4 ± 9.1 years) than those in the ALT ≤30 group (52.4 ± 9.3 years). Those with elevated ALT levels also had higher body mass index (26.5 ± 4.2 vs 23.4 ± 3.1 kg/m^2^) and waist circumference (91.1 ± 10.3 vs 82.5 ± 8.9 cm) than participants with lower ALT levels. Current smoking and drinking were slightly more prevalent in the ALT >30 group, with a marginally higher weekly alcohol intake.

As shown in Table 2, the prevalence of FIB-4 values exceeding 1.30, 2.01, and 2.67 increased with age in the total population and in participants with ALT >30 IU/L. In the total population, FIB-4 exceeded the cutoff of 1.30 in 23.0% of participants, whereas it exceeded the thresholds of 2.01 and 2.67 in 3.8% and 0.9% of participants, respectively. The prevalence of FIB-4 was notably higher in the older age groups. In contrast, FIB-3 values ≥3.41 remained relatively stable, with an overall prevalence of 1.3%, and did not show a large increase with age. Among participants with ALT levels >30 IU/L, 21.1% exceeded the FIB-4 cutoff of 1.30, whereas 4.4% and 1.7% exceeded the thresholds of 2.01 and 2.67, respectively. The prevalence of FIB-3 ≥ 3.41 was higher in this group, at 3.3%, but did not substantially increase with age.

Figure 2 shows a scatter plot of FIB-4 versus FIB-3, with data points color-coded according to age group. The plot reveals a clear trend: as FIB-3 values increased, FIB-4 also increased, particularly in older age groups. This upward relationship between FIB-3 and FIB-4 was most evident in participants in their 50s and 60s, in whom higher FIB-3 values corresponded to a noticeable increase in FIB-4 levels.

Table 3 shows the agreement between the FIB-3 and FIB-4 cutoff values among participants with ALT levels of >30 IU/L. The results show consistently high NPVs for FIB-3 ≥ 3.41 across various FIB-4 cutoff values (1.30, 2.01, and 2.67) and age groups. For FIB-4 ≥ 1.30, the NPV was 99.9%, indicating that participants with FIB-3 values <3.41 were highly unlikely to have FIB-4 values >1.30. Both PABAK and AC1 values demonstrated good agreement between the FIB-3 and FIB-4 cutoff values in most age groups, with PABAK generally ranging from 0.64 to 0.96 and AC1 ranging from 0.77 to 0.98, indicating similar trends. In the 60s age group for FIB-4 ≥ 1.30, both PABAK and AC1 indicated poor agreement; however, overall, the values suggested strong concordance between FIB-3 and FIB-4 measures. These results are presented only for participants with ALT levels >30 IU/L, as the findings were consistent with those observed in the total population (Table S2). Additional analysis for women (Table S3) demonstrated similar trends, further supporting the robustness of the results across sexes.

Table 4 shows the factors associated with false-positive FIB-3 results (FIB-3 ≥ 3.41) despite negative FIB-4 outcomes (FIB-4 < 2.67) among the participants with ALT levels >30 IU/L. Age was positively associated with false-positive FIB-3 results (OR: 1.05; 95% CI, 1.01-1.09; *P* = .03). Among biochemical variables, AST levels were significantly associated with false positives (OR: 1.12; 95% CI, 1.10-1.14: *P* < .01). Higher alcohol intake (≥140 g/wk) was also associated with increased odds of false-positive FIB-3 results (OR: 5.07; 95% CI, 1.37-18.7; *P* = .03), as well as alcohol intake per 20 g/wk increase (OR: 1.18; 95% CI, 1.04-1.33; *P* = .01). Other factors, such as body mass index, waist circumference, and GGT levels, showed no significant associations. The findings were consistent with those observed in the total population (Table S4).

**Table 4:** Factors associated with false-positive FIB-3 results (FIB-3 ≥ 3.41) despite negative FIB-4 outcomes (FIB-4 < 2.67) among participants with ALT >30 (*n* = 3623).

**Variable**	**β**	**SE**	**OR**	**(95% CI)**	***P* value**
Age	.05	0.02	1.05	(1.01-1.09)	.03
Male	−.29	0.39	0.56	(0.12-2.62)	.46
BMI	−.08	0.10	0.92	(0.75-1.13)	.42
Waist	−.01	0.04	0.99	(0.91-1.07)	.77
AST	.11	0.01	1.12	(1.10-1.14)	<.01
GGT	.01	0.01	1.00	(1.00-1.01)	.12
Alcohol intake, g/wk	None (reference)				
0 to <70	−.01	0.27	2.10	(0.80-5.50)	.97
70 to <140	−.11	0.31	1.91	(0.64-5.74)	.73
<140	.87	0.40	5.07	(1.37-18.7)	.03
per 20 g/wk increase (continuous)	.16	0.06	1.18	(1.04-1.33)	.01

Abbreviations: ALT, alanine aminotransferase; AST, aspartate aminotransferase; BMI, body mass index; GGT, γ-glutamyl transferase; OR, odds ratio.

## 4. Discussion

In this study, we evaluated the potential utility of FIB-3 as a secondary screening tool following general health checkups by examining the concordance and discrepancies between FIB-4 and FIB-3 in participants with ALT levels >30 IU/L and identifying factors contributing to these discrepancies. Overall, FIB-3 demonstrated consistently high NPVs, especially in younger participants, in whom the concordance between FIB-3 and FIB-4 was strong, as indicated by high agreement statistic values. This suggests that incorporating FIB-3, which relies on platelet counts obtained during routine health checkups, can avoid unnecessary thorough examinations following positive health checkup results. Additionally, physicians could consider integrating FIB-3 results with drinking history or GGT levels to better identify alcohol-related liver injury and prioritize referrals for further evaluation. Furthermore, discrepancies between FIB-3 and FIB-4 were influenced by factors such as alcohol consumption, emphasizing the relevance of FIB-3 in evaluating liver fibrosis irrespective of etiology.

The selection of appropriate cutoff values is crucial for both FIB-3 and FIB-4. In this study, we used commonly applied clinical cutoff values of 1.30 and 2.67 for FIB-4 and compared them with the FIB-3 cutoff value of 3.41.^12^^,^^17^ The concordance between FIB-4 ≥ 1.30 and FIB-3 ≥ 3.41 was high across most age groups, with AC1 values ranging from 0.73 to 1.00 in participants aged 30 to 50 years. The inherent age sensitivity of FIB-4, with a tendency for false negatives in younger individuals and false positives in older individuals, presents challenges for its application in health checkups for working populations. By excluding age from its formula, FIB-3 mitigates these limitations and provides a more stable assessment of fibrosis across age groups. For FIB-4 ≥ 2.01 and FIB-3 ≥ 3.41, concordance was higher in the 60s group, with AC1 values reaching 0.90. Concordance between FIB-4 ≥ 2.67 and FIB-3 ≥ 3.41 was the highest, with AC1 values ranging from 0.97 to 0.98 across all age groups. These findings suggest a strong alignment between FIB-3 and FIB-4 at higher cutoff values, particularly among older participants, indicating that both markers may be useful for identifying individuals at risk of fibrosis.

To further evaluate the utility of FIB-3 in the context of the recent EASL-EASD-EASO guidelines,^23^ we analyzed the proportion of individuals excluded from referral pathways using the FIB-3 (≥3.41). Among individuals with FIB-4 of 1.3-2.67, 94.2% (2731/2781) could be excluded using FIB-3. For individuals ≥65 years with FIB-4 ≥ 2.0, 89.1% (155/174) were excluded. In the total population with FIB-4 ≥ 2.67, 27.4% (32/117) could be excluded. These findings demonstrate the substantial value of FIB-3 in reducing unnecessary referrals, particularly for individuals in the lower FIB-4 range (1.3-2.67) and older adults (≥65 years with FIB-4 ≥ 2.0). Even among those in the highest FIB-4 category (≥2.67), FIB-3 could exclude a significant proportion, supporting its role in streamlining referral decisions and optimizing health care resource allocation.

Recent changes in workstyles have led to increased sitting time and concerns about a decline in overall physical activity,^24^ contributing to an increased risk of fatty liver disease alongside obesity-induced metabolic syndrome.^25^ As a result, the proportion of workers exhibiting abnormal liver function during health checkups is expected to increase. Although only a small proportion of individuals with liver disease progress to advanced fibrosis or hepatocellular carcinoma, identifying effective screening methods is a critical public health issue.^26^^,^^27^ In this study, 28.7% of the participants had ALT levels of >30 IU/L, the threshold proposed by the Japan Society of Hepatology, which indicates a high risk of fatty liver disease.^5^ Identifying patients at risk of progression to fibrosis is essential. In population-based health checkups, particularly for working populations with a significant proportion of younger individuals, the NPV of fibrosis markers is more critical than the positive predictive value. A high NPV ensures that individuals without fibrosis are effectively excluded from further evaluation, addressing the limitations of FIB-4 in younger participants where false negatives may occur. In this study, assuming FIB-4 > 2.01 indicates true fibrosis, the NPV of FIB-3 was 99.4% (3444/3465). When using FIB-4 > 2.67 as the cutoff for true fibrosis, the NPV of FIB-3 was 98.2% (3498/3561). Additional analysis restricted to women showed similar trends, confirming that the inclusion of both men and women does not affect the results. These findings, consistent across sexes, suggest that FIB-3 ≥ 3.41 can be effectively used for secondary screening following general health checkups.

The FIB-3 index was developed to address the issue of elevated FIB-4 levels in older individuals.^11^^,^^12^ Despite being designed to mitigate the age-related elevation seen in FIB-4, FIB-3 showed strong agreement with FIB-4 in older age groups, mainly when using the higher FIB-4 cutoff (≥2.67). This suggests that older individuals with FIB-3 and FIB-4 values above their respective cutoffs may be at a significantly higher risk of fibrosis and should be prioritized for further evaluation. However, our findings also showed that older age was significantly associated with false-positive FIB-3 results, possibly due to age-related increases in AST and decreases in platelet counts—both components of the FIB-3 formula. The concordance between FIB-3 and FIB-4 was high in younger participants, suggesting that FIB-3 effectively rules out fibrosis. By excluding age as a factor, FIB-3 avoids the false negatives associated with FIB-4 in younger individuals, making it particularly suitable for health checkups targeting working populations. FIB-3 may also help to detect liver fibrosis, particularly in cases where FIB-4 exceeds 2.67, and may have a higher positive predictive value in younger individuals. Our results showed that elevated FIB-3 levels in younger participants were associated with high AST levels and significant alcohol consumption, suggesting that FIB-3 is sensitive to liver damage irrespective of its etiology. An analysis restricted to nondrinkers showed similar associations, indicating that these findings were not driven solely by alcohol intake. However, in high-risk individuals with heavy alcohol use or elevated GGT, additional markers or imaging modalities, such as transient elastography, may help refine screening accuracy and reduce the risk of false positives. This is consistent with prior findings that FIB-4 is a good indicator of liver damage, including alcohol-induced injury, in individuals with elevated GGT levels,^13^ further highlighting the role of FIB-3 in detecting liver fibrosis caused by various factors.

The strengths of this study include the use of a large cohort of workers and both FIB-3 and FIB-4 levels, allowing for a comprehensive comparison of these fibrosis markers. However, this study has several limitations. This was a cross-sectional study, which limited our ability to examine causality or track the progression of liver disease over time. Additionally, as the study population was derived from a specific corporate organization, the findings may be influenced by unique characteristics of this group, such as occupation, industry, or lifestyle factors, potentially limiting their generalizability. Future studies should include more diverse populations from various occupational settings to validate the utility of FIB-3 in broader contexts and ensure its applicability across different demographic and workplace environments. Furthermore, the outcome was not based on pathological confirmation, indicating that the true diagnostic accuracy of FIB-3 and FIB-4 in detecting fibrosis remains uncertain. Although we assessed the concordance between these markers, future studies should evaluate their NPV in relation to actual pathological findings to better determine their clinical utility.

## 5. Conclusions

This study demonstrated the strong concordance of FIB-3 and FIB-4, particularly in individuals with ALT levels of >30 IU/L. FIB-3 showed a consistently high NPV across various FIB-4 cutoff values, suggesting its utility as a secondary screening tool for liver fibrosis in general health checkups, regardless of etiology. In clinical practice, high sensitivity and positive predictive value are often prioritized. However, in the context of general health examinations, a high NPV is crucial for reducing unnecessary follow-ups. Although the results highlight the potential of FIB-3 in reducing unnecessary thorough examinations, future research should validate these findings against pathological outcomes to support the incorporation of FIB-3 and platelet count into routine health assessments.

## Supplementary Material

Web_Material_uiaf038

## Data Availability

The datasets used in this study are not publicly available due to restrictions under the license for the current study. These are available on reasonable request from the corresponding author.
