# Supplementary material for: Comparative evaluation of FIB-3 and FIB-4 indices for liver fibrosis screening in workplace-based health checkups
Source: J Occup Health. 2025 Jul 9;67(1):uiaf038. doi: 10.1093/joccuh/uiaf038 (PMC12341935; doi:10.1093/joccuh/uiaf038)
Supplement: Web_Material_uiaf038 [file web_material_uiaf038.zip › Supplement_2025-07-07.docx]

**Supplementary Table 1. STROBE Statement for observational studies**

|  | Item No. | | Recommendation | Page  No. |
| --- | --- | --- | --- | --- |
| **Title and abstract** | 1 | | (*a*) Indicate the study’s design with a commonly used term in the title or the abstract | Title page |
|  |  |  | (*b*) Provide in the abstract an informative and balanced summary of what was done and what was found | 1,2 |
| Introduction | | | | |
| Background/rationale | 2 | | Explain the scientific background and rationale for the investigation being reported | 3,4 |
| Objectives | 3 | | State specific objectives, including any pre-specified hypotheses | 4 |
| Methods | | | | |
| Study design | 4 | | Present key elements of study design early in the paper | 4,5 |
| Setting | 5 | | Describe the setting, locations, and relevant dates, including periods of recruitment, exposure, follow-up, and data collection | 4,5 |
| Participants | 6 | | (*a*) *Cohort study*—Give the eligibility criteria, and the sources and methods of selection of participants. Describe methods of follow-up | 5 |
|  |  |  | (*b*) *Cohort study*—For matched studies, give matching criteria and number of exposed and unexposed | n/a |
| Variables | 7 | | Clearly define all outcomes, exposures, predictors, potential confounders, and effect modifiers. Give diagnostic criteria, if applicable | 6,7 |
| Data sources/ measurement | 8* | | For each variable of interest, give sources of data and details of methods of assessment (measurement). Describe comparability of assessment methods if there is more than one group | 6,7 |
| Bias | 9 | | Describe any efforts to address potential sources of bias | 7 |
| Study size | 10 | | Explain how the study size was arrived at | 5 |
| Quantitative variables | 11 | | Explain how quantitative variables were handled in the analyses. If applicable, describe which groupings were chosen and why | 6,7,8 |
| Statistical methods | 12 | | (*a*) Describe all statistical methods, including those used to control for confounding | 6,7,8 |
|  |  |  | (*b*) Describe any methods used to examine subgroups and interactions | 7 |
|  |  |  | (*c*) Explain how missing data were addressed | n/a |
|  |  |  | (*d*) *Cohort study*—If applicable, explain how loss to follow-up was addressed | n/a |
|  |  |  | (*e*) Describe any sensitivity analyses | 7 |
| Participants | 13* | | (a) Report numbers of individuals at each stage of study—e.g. numbers potentially eligible, examined for eligibility, confirmed eligible, included in the study, completing follow-up, and analyzed | 8 |
|  |  |  | (b) Give reasons for non-participation at each stage | n/a |
|  |  |  | (c) Consider use of a flow diagram | Figure 1. |
| Descriptive data | 14* | | (a) Give characteristics of study participants (e.g. demographic, clinical, social) and information on exposures and potential confounders | Table 1, |
|  |  |  | (b) Indicate number of participants with missing data for each variable of interest | n/a |
|  |  |  | (c) *Cohort study*—summarize follow-up time (e.g., average and total amount) | n/a |
| Outcome data | 15* | | *Cohort study*—Report numbers of outcome events or summary measures over time | 8 |
| Main results | 16 | | (*a*) Give unadjusted estimates and, if applicable, confounder-adjusted estimates and their precision (eg, 95% confidence interval). Make clear which confounders were adjusted for and why they were included | Table 2,3,4 |
|  |  |  | (*b*) Report category boundaries when continuous variables were categorized | n/a |
|  |  |  | (*c*) If relevant, consider translating estimates of relative risk into absolute risk for a meaningful time period | - |
| Other analyses | 17 | Report other analyses done—e.g. analyses of subgroups and interactions, and sensitivity analyses | | 9-10 |
| Key results | 18 | Summarize key results with reference to study objectives | | 12 |
| Limitations | 19 | Discuss limitations of the study, taking into account sources of potential bias or imprecision. Discuss both direction and magnitude of any potential bias | | 10 |
| Interpretation | 20 | Give a cautious overall interpretation of results considering objectives, limitations, multiplicity of analyses, results from similar studies, and other relevant evidence | | 12,13 |
| Generalizability | 21 | Discuss the generalizability (external validity) of the study results | | 12,13 |
| Other information | |  | |  |
| Funding | 22 | Give the source of funding and the role of the funders for the present study and, if applicable, for the original study on which the present article is based | | Title page |

**Supplementary Table 2.** Agreement for different FIB-4 cutoffs compared to FIB-3≥3.41 by age-class among total participants (N=12,622).

|  |  | FIB-4≥1.30 | |  |  |  |  | FIB-4≥2.01 | |  |  |  |  | FIB-4≥2.67 | |  |  |  |
| --- | --- | --- | --- | --- | --- | --- | --- | --- | --- | --- | --- | --- | --- | --- | --- | --- | --- | --- |
|  |  | (-) | (+) | PABAK | AC1 | NPV |  | (-) | (+) | PABAK | AC1 | NPV |  | (-) | (+) | PABAK | AC1 | NPV |
| Age class | FIB-3 ≥3.41 | N | N | (95% CI) | (95% CI) |  |  | N | N | (95% CI) | (95% CI) |  |  | N | N | (95% CI) | (95% CI) |  |
| 30s | (-) | 1423 | 13 | 0.98 | 0.99 | 99.1% |  | 1436 | 0 | 1.00 | 1.00 | 100.0% |  | 1436 | 0 | ― | ― | 100.0% |
|  | (+) | 1 | 3 | (0.97–0.99) | (0.99–1.00) |  |  | 3 | 1 | (0.99–1.00) | (1.00–1.00) |  |  | 4 | 0 | ― | ― |  |
| 40s | (-) | 3310 | 169 | 0.90 | 0.95 | 95.1% |  | 3478 | 1 | 0.99 | 1.00 | 100.0% |  | 3479 | 0 | 0.99 | 0.99 | 100.0% |
|  | (+) | 0 | 31 | (0.89–0.92) | (0.94–0.96) |  |  | 15 | 16 | (0.99–1.00) | (0.99–1.00) |  |  | 24 | 7 | (0.98–0.99) | (0.99–1.00) |  |
| 50s | (-) | 3755 | 984 | 0.59 | 0.74 | 79.2% |  | 4688 | 51 | 0.98 | 0.99 | 98.9% |  | 4735 | 4 | 0.98 | 0.99 | 99.9% |
|  | (+) | 0 | 71 | (0.57–0.61) | (0.72–0.76) |  |  | 5 | 66 | (0.97–0.98) | (0.98–0.99) |  |  | 41 | 30 | (0.98–0.99) | (0.99–0.99) |  |
| 60s | (-) | 1235 | 1565 | -0.09 | 0.06 | 44.1% |  | 2519 | 281 | 0.80 | 0.89 | 90.0% |  | 2772 | 28 | 0.97 | 0.98 | 99.0% |
|  | (+) | 0 | 62 | (-0.13–-0.06) | (0.02–0.11) |  |  | 0 | 62 | (0.78–0.83) | (0.87–0.90) |  |  | 14 | 48 | (0.96–0.98) | (0.98–0.99) |  |
| Total | (-) | 9723 | 2731 | 0.57 | 0.72 | 78.1% |  | 12121 | 333 | 0.94 | 0.97 | 97.3% |  | 12422 | 32 | 0.98 | 0.99 | 99.7% |
|  | (+) | 1 | 167 | (0.55–0.58) | (0.71–0.74) |  |  | 23 | 145 | (0.94–0.95) | (0.97–0.97) |  |  | 83 | 85 | (0.98–0.99) | (0.99–0.99) |  |

Abbreviations: AC1, Gwet’s AC1 statistic; NPV, negative predictive value; PABAK, Prevalence-adjusted Bias-adjusted Kappa.

**Supplementary Table 3.** Agreement for different FIB-4 cutoffs compared to FIB-3≥3.41 by age-class among female participants (N=1,644).

|  |  | FIB-4≥1.30 | |  |  |  |  | FIB-4≥2.01 | |  |  |  |  | FIB-4≥2.67 | |  |  |  |
| --- | --- | --- | --- | --- | --- | --- | --- | --- | --- | --- | --- | --- | --- | --- | --- | --- | --- | --- |
|  |  | (-) | (+) | PABAK | AC1 | NPV† |  | (-) | (+) | PABAK | AC1 | NPV |  | (-) | (+) | PABAK | AC1 | NPV |
| Age class | FIB-3 ≥3.41 | N | N | (95% CI) | (95% CI) |  |  | N | N | (95% CI) | (95% CI) |  |  | N | N | (95% CI) | (95% CI) |  |
| 30s | (-) | 120 | 0 | ― | ― | 100.0% |  | 120 | 0 | ― | ― | 100.0% |  | 120 | 0 | ― | ― | 100.0% |
|  | (+) | 0 | 0 | ― | ― |  |  | 0 | 0 | ― | ― |  |  | 0 | 0 | ― | ― |  |
| 40s | (-) | 398 | 18 | 0.91 | 0.95 | 100.0% |  | 416 | 0 | 1.00 | 1.00 | 100.0% |  | 416 | 0 | 1.00 | 1.00 | 100.0% |
|  | (+) | 0 | 2 | (0.88–0.95) | (0.93–0.98) |  |  | 0 | 2 | (1.00–1.00) | (1.00–1.00) |  |  | 0 | 2 | (1.00–1.00) | (1.00–1.00) |  |
| 50s | (-) | 435 | 156 | 0.48 | 0.66 | 100.0% |  | 579 | 12 | 0.96 | 0.98 | 100.0% |  | 590 | 1 | 0.99 | 0.99 | 99.5% |
|  | (+) | 0 | 5 | (0.41–0.55) | (0.60–0.71) |  |  | 0 | 5 | (0.94–0.98) | (0.97–0.99) |  |  | 3 | 2 | (0.97–1.00) | (0.99–1.00) |  |
| 60s | (-) | 226 | 279 | -0.09 | 0.01 | 100.0% |  | 452 | 53 | 0.79 | 0.88 | 100.0% |  | 502 | 3 | 0.98 | 0.99 | 99.8% |
|  | (+) | 0 | 5 | (-0.18–-0.01) | (-0.02–0.18) |  |  | 0 | 5 | (0.74–0.85) | (0.85–0.92) |  |  | 1 | 4 | (0.97–1.00) | (0.98–1.00) |  |
| Total | (-) | 1179 | 453 | 0.45 | 0.63 | 100.0% |  | 1567 | 65 | 0.92 | 0.96 | 100.0% |  | 1628 | 4 | 0.99 | 1.00 | 99.8% |
|  | (+) | 0 | 12 | (0.41–0.49) | (0.60–0.67) |  |  | 0 | 12 | (0.90–0.94) | (0.95–0.97) |  |  | 4 | 8 | (0.98–1.00) | (0.99–1.00) |  |

Abbreviations: AC1, Gwet’s AC1 statistic; NPV, negative predictive value; PABAK, Prevalence-adjusted Bias-adjusted Kappa.

**Supplementary Table 4.** Factors associated to false positive FIB-3 results (FIB-3≥3.41) despite negative FIB-4 outcomes (FIB-4<2.67) among total participants (N=12,622).

| Variable | β | SE | OR | (95% CI) | p-value |
| --- | --- | --- | --- | --- | --- |
| Age | 0.03 | 0.02 | 1.03 | (1.00–1.07) | 0.06 |
| Male | 0.27 | 0.37 | 1.71 | (0.41–7.18) | 0.46 |
| BMI | -0.03 | 0.09 | 0.97 | (0.81–1.17) | 0.77 |
| Waist | -0.06 | 0.03 | 0.94 | (0.88–1.01) | 0.08 |
| AST | 0.13 | 0.01 | 1.14 | (1.12–1.16) | <.01 |
| GGT | 0.00 | 0.00 | 1.00 | (1.00–1.00) | 0.46 |
| Alcohol intake, g/week | None (reference) | |  |  |  |
| 0< to 70 | -0.18 | 0.23 | 2.50 | (1.03–6.11) | 0.43 |
| 70< to 140 | 0.18 | 0.25 | 3.59 | (1.37–9.38) | 0.47 |
| <140 | 1.11 | 0.32 | 9.09 | (2.97–27.85) | <.01 |
| per 20 g/week increase (continuous) | 0.22 | 0.05 | 1.24 | (1.13–1.37) | <.01 |

Abbreviations: CI, confidence interval; OR, odds ratio; SE, standard error.
